# Effect of the inserted active-site-covering lid loop on the catalytic activity of a mutant *B. subtilis* γ-glutamyltransferase (GGT)†

**DOI:** 10.1039/c9ra05941e

**Published:** 2019-10-28

**Authors:** Michela Massone, Cinzia Calvio, Marco Rabuffetti, Giovanna Speranza, Carlo F. Morelli

**Affiliations:** Department of Chemistry, Università degli Studi di Milano Via Golgi, 19 20133 Milano Italy carlo.morelli@unimi.it; Department of Biology and Biotechnology, Università degli Studi di Pavia Via Ferrata, 9 27100 Pavia Italy; Dipartimento di Scienze per gli Alimenti, la Nutrizione e l’Ambiente, Università degli Studi di Milano Via Mangiagalli, 25 20133 Milano Italy; Istituto di Scienze e Tecnologie Molecolari (INSTM), CNR, c/o Department of Chemistry Via Golgi, 19 20133 Milano Italy

## Abstract

γ-Glutamylpeptides are compounds derived from the acylation of an amino acid or a short peptide by the γ-carboxyl carbon of the side chain of glutamic acid. Due to their altered chemico-physical and organoleptic properties, they may be interesting substitutes or precursors of parent compounds used in pharmaceutical, dietetic and cosmetic formulations. Some of them are naturally occurring flavor enhancers or are endowed with biological activities. Enzymatic approaches to the synthesis of γ-glutamyl derivatives based on the use of γ-glutamyltransferases (GGTs, EC 2.3.2.2) have been proposed, which should be able to alleviate the problems connected with the troublesome and low-yielding extraction from natural sources or the non-economical chemical synthesis, which requires protection/deprotection steps. With the aim of overcoming the current limitations in the use of GGTs as biocatalysts, a mutant GGT was investigated. The mutant GGT was obtained by inserting the active-site-covering lid loop of the *E. coli* GGT onto the structure of *B. subtilis* GGT. With respect to the wild-type enzyme, the mutant showed a more demanding substrate specificity and a low hydrolase activity. These results represent an attempt to correlate the structural features of a GGT to its different activities. However, the ability of the mutant enzyme to catalyze the subsequent addition of several γ-glutamyl units, inherited by the parent *B. subtilis* GGT, still represents a limitation to its full application as a biocatalyst for preparative purposes.

## Abstract 2

A mutant γ-glutamyltransferase with improve transpeptidase activity was obtained by inserting the active site-covering lid loop on an enzyme naturally lacking it.

## Graphical abstract

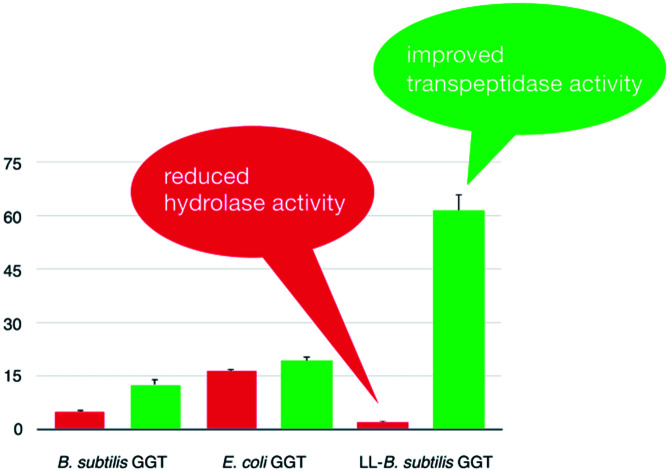

## 1. Introduction

**Fig. 1:**
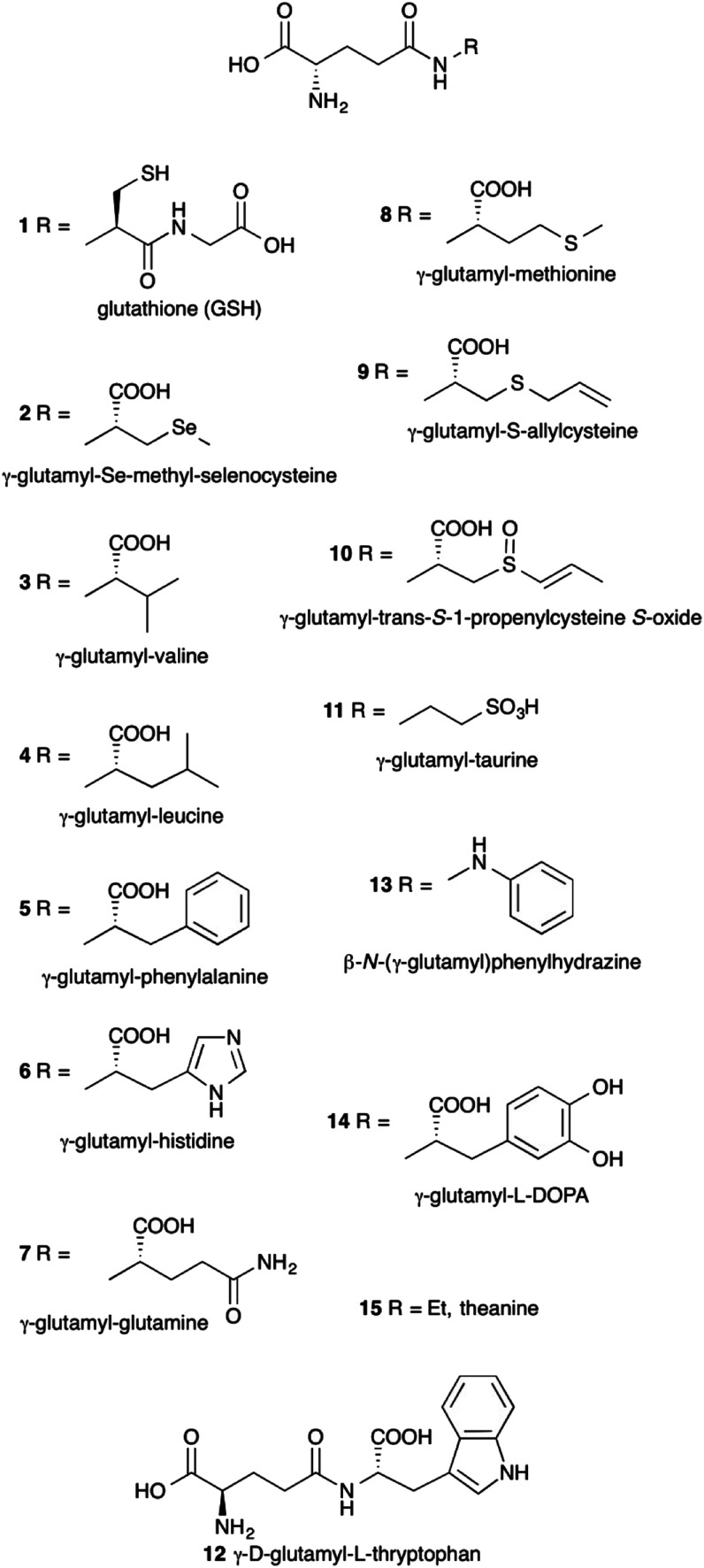
Examples of γ-glutamyl derivatives.

γ-Glutamyl compounds (Fig. 1) are defined as being derived by the acylation of an amino acid or a short peptide through the γ-carboxyl carbon of a glutamic acid moiety. The prototypical γ-glutamyl derivative is glutathione (γ-glutamyl-cysteinyl-glycine, GSH) 1. With respect to parent compounds, γ-glutamyl derivatives exhibit different physico-chemical and organoleptic properties. For example, since the γ-glutamyl moiety introduced has polar functional groups, the solubility of γ-glutamyl compounds in water may be enhanced compared to that of the underivatized amino acids.^1^ γ-Glutamyl-*Se*-methyl-seleno-cysteine 2 represents a convenient source of the micronutrient selenium, free of the unpleasant smell but equally effective in its biological activity as *Se*-methyl-selenocysteine.^2^ Also, the bitter taste of branched-chain and aromatic amino acids 3–5 is severely reduced upon γ-glutamylation.^3^

γ-Glutamyl derivatives are also widespread in nature. Glutathione 1 represents the most abundant thiol in cells. Several γ-glutamyl compounds, including glutathione itself, are taste-active compounds with kokumi properties.^4,5^ Kokumi is a Japanese term that refers to gustative sensations related to mouthfulness, roundness and continuity. Kokumi compounds are naturally occurring flavor enhancers^6^ tasteless by themselves but able to elicit their activity in conjunction with other taste-active components in food through the interaction with a Calcium sensing receptor (CaSR).^7,8^ γ-Glutamyl derivatives with kokumi properties have been isolated from several foods, *e.g.* compounds 3–8 among others,^9–12^ and they are characteristic constituents of edible vegetables (3 and 4),^13^ especially those commonly used as seasoning ingredients in worldwide cuisine, such as garlic (9)^14^ and onion (10).^15^ In addition, some of such γ-glutamyl derivatives (8 and 10) show interesting biological activities,^16,17^ which lead to considering them as nutraceuticals.

Despite their relatively simple chemical structure, a practical transformation into γ-glutamyl derivatives is not a trivial task. The enzymatic approach has been envisaged as a more convenient preparation method with respect to both the laborious and low-yielding extractions from natural sources and the synthesis through the classical peptide chemistry, plagued by the need of protection–deprotection steps.

Enzymatic synthesis of γ-glutamyl derivatives can be tackled by the use of synthetases such as γ-glutamylmethylamide synthetase (EC 6.3.4.12),^18^ which however requires an ATP-supplying and recycling system.

Despite the use of glutaminases (EC 3.5.1.2) has also been recently proposed,^17,19–21^ most of the enzymatic synthesis of γ-glutamyl compounds relies on the use of a γ-glutamyltransferase (GGT, EC 2.3.2.2) as the biocatalyst.^2,22–42^

**Scheme 1:**
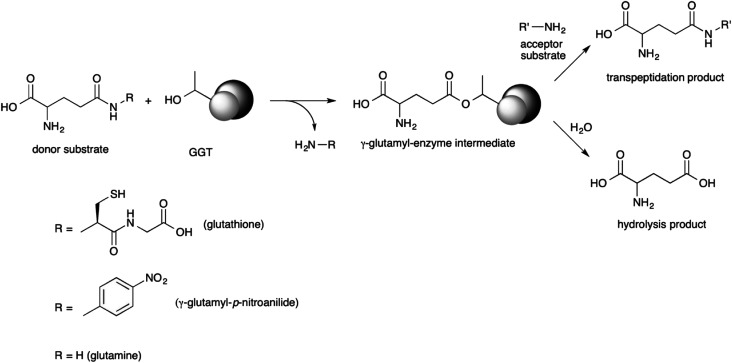
γ-glutamyltransferase-catalyzed reactions.

GGT is a heterodimeric enzyme arising from a post-translational cleavage of a single polypeptide chain into a small and a large subunit.^43–45^ It is mainly involved in glutathione metabolism^46^ and is highly conserved from bacteria to plants and animals.^47^ It cleaves glutathione, or other γ-glutamyl donors, transiently binding the γ-glutamyl moiety in a γ-glutamyl-enzyme intermediate through an ester bond formed by the hydroxyl group of the catalytically active threonine residue, located at the N-terminal end of the small enzyme subunit.^48,49^ The γ-glutamyl residue is then either transferred to a water molecule in a hydrolysis reaction, or to an amino acid or a short peptide in a transpeptidation reaction, leading to a new γ-glutamyl derivative (Scheme 1).^50^ The extent of the hydrolase and transpeptidase activities exerted by GGTs is usually pH-dependent, with basic pHs promoting the transpeptidation reaction over the hydrolysis. The optimum pH of the reaction medium for transpeptidation depends on the p*K*_a_ of the amino group of the acceptor substrate, which should be in the unprotonated form in order to be competent as a nucleophile.^51,52^ Although the transpeptidation-to-hydrolysis ratio for mammalian GGT is usually higher and better controlled through pH adjustment,^53^ bacterial GGTs are preferred as biocatalysts for preparative purposes. They are easily produced and purified,^41,54–58^ avoiding the risk of viral contamination associated with an enzyme isolated from an animal organ. In addition, glutamine can be used as a less expensive γ-glutamyl-donor substrate with respect to glutathione, required by GGTs from higher organisms.^35,54,56^

The GGT from *E. coli* (EcGGT) has been extensively used for preparative purposes. The synthesis of γ-glutamylvaline 3,^29^ γ-glutamylphenylalanine 5 (ref. 3) and γ-glutamylglutamine 7 (ref. 32) has been employed to obtain tasteless precursors of the bitter parent amino acids. Biologically active compounds such as γ-glutamyltaurine 11,^27^ γ-d-glutamyl-l-tryptophan 12 (ref. 30) and β-*N*-(γ-l-glutamyl)phenylhydrazine 13,^34^ or prodrugs such as γ-glutamyl-l-DOPA 14 (ref. 23) have also been prepared using EcGGT as biocatalyst. A common byproduct of EcGGT-catalyzed reactions is γ-glutamylglutamine 7, arising from a so-called autotranspeptidation reaction, *i.e.* a reaction in which the same compound acts as both the donor and the acceptor substrate.

In addition to EcGGT, some GGTs from *Bacillus* spp. have been proposed as biocatalysts. An evident structural feature that characterizes the GGT of *B. subtilis* (BsGGT) is the lack of a sequence, called lid loop, that covers the γ-glutamyl binding site in most of the known GGTs.^59^

The synthesis of the amino acid theanine 15 was accomplished using GGTs from different strains of *B. subtilis*.^33,36,38^ γ-Glutamyl-*S*-allylcysteine 9,^39^ γ-glutamylphenylalanine 5 (ref. 40) and γ-glutamylleucine 4 (ref. 42) were obtained through reactions catalyzed by the GGT from *B. licheniformis*, while GGT from *B. atrophaeus* was used for the enzymatic synthesis of γ-d-glutamyl-l-tryptophan 12.^41^

Although attractive for their simplicity and for the lack of required protection/deprotection steps, in general the enzymatic approaches to the synthesis of γ-glutamyl derivatives suffer from rather low yields and formation of byproducts from autotranspeptidation (γ-glutamylglutamine) and hydrolysis reactions (glutamic acid).

**Fig. 2:**
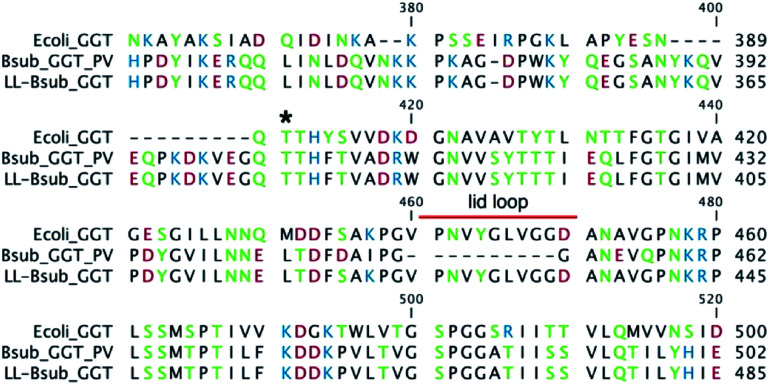
Excerpt of the sequence alignment of *E. coli* GGT (upper line), *B. subtilis* GGT (middle line) and the mutant LL-BsGGT (lower line). The catalytically active N-terminal Thr residue of the small subunit is marked with *. The sequence of the lid loop starts after residue 460 (overall numbering).

We recently obtained a mutant GGT by inserting the lid loop of EcGGT onto the structure of BsGGT and we called this enzyme LL-BsGGT (from Lid Loop *B. subtilis* GGT) (Fig. 2). Through the use of synthetic oligo-γ-glutamylglutamines as model donor compounds in hydrolysis reactions, we concluded that the presence of the lid loop is able to affect donor substrate selection on the basis of molecular size.^60^ In this work, the promising features of LL-BsGGT have been elucidated, demonstrating that the recombinant enzyme shows enhanced tranpeptidase activity with respect to its wt (wild-type) counterpart. LL-BsGGT then could have potentialities as a biocatalyst for the enzymatic synthesis of γ-glutamyl derivatives with kokumi properties.

## 2. Experimental

### 2.1 General

l-Glutamine, l-glutamic acid, l-serine, glycilglycine (GlyGly), l-glutamic acid 5-(*p*-nitroanilide) (GPNA) and 1-fluoro-2,4-dinitrobenzene (Sanger's reagent) were from Sigma Aldrich (Darmstadt, Germany); l-alanylglycine, l-2-aminobutrylglycine (Abu-Gly) and l-leucylglycyne were from Bachem (Bubendorf, Switzerland). All reagents were used as received without further purification.

l-valylglycine and γ-glutamylvalylglicine were prepared as reported in ESI.†*S*-Allylcysteine and γ-glutamyl-*S*-allylcysteine were prepared as previously described.^52^

HPLC-grade solvents were from Aldrich.

Analytical TLC was performed on silica gel F_254_ pre-coated aluminum sheets (0.2 mm layer) (Merck, Darmstadt, Germany). Elution solvents are reported for each specific product's preparation. Detection: UV lamp (*λ* 254 nm), 4.5% w/v CeSO_4_/(NH_4_)_6_Mo_7_O_24_·4H_2_O solution or 5% w/v ninhydrin solution in ethanol, followed by heating at 150 °C *ca.*

HPLC analyses were carried out with a Jasco instrument equipped with UV/Vis detector, using a 250 × 4.6 mm Gemini RP C18 column (Phenomenex, Torrance, CA, USA). Eluent A was 0.1% trifluoroacetic acid; eluent B was a 80 : 20 mixture of acetonitrile and eluent A. The compounds, derivatized with Sanger's reagent, were analyzed with the following gradient: 0–10 min, eluent A : eluent B 80 : 20 isocratic elution; 10–15 min, linear gradient to eluent A : eluent B 70 : 30; 15–25 min, linear gradient to eluent A : eluent B 40 : 40; 25–35 min, linear gradient to eluent A : eluent B 40 : 60; 35–40 min, isocratic elution eluent A : eluent B 40 : 60; 40–60 min, column riequilibration through linear gradient to eluent A eluent B 80 : 20. Flow rate was 1 mL min^−1^ and detection was at 356 nm.

Ion exchange chromatography was performed with Dowex 1 × 8 resin 200–400 mesh (Aldrich, Darmstadt, Germany) in the acetate form. Resin was equilibrated before use with 5 volumes of 0.5 M acetic acid.

^1^H-NMR and ^13^C-NMR spectra were acquired at 400.13 MHz and 100.61 MHz, respectively, on a Bruker Advance 400 spectrometer (Bruker, Karlsruhe, Germany) interfaced with a workstation running Windows operating system and equipped with a TOPSPIN software package. ^13^C signal multiplicities were based on attached proton test experiments (APT) and attributions were based on HSQC (Hetero Single Quantum Correlation) and HMBC (Hetero Multiple Bond Correlation) experiments. Chemical shifts are given in ppm (*δ*) and are referenced to solvent signal (*δ*_H_ D_2_O 4.79 ppm) or to TSP (3-(trimethylsilyl)propionic-2,2,3,3-d4 acid sodium salt) as external standard (*δ*_Me_ 0.00 ppm). Spectra analyses were carried out with inmr Reader software (http://www.inmr.net).

ESI-MS spectra were recorded on a Thermo Finnigan LCQ Advantage spectrometer (Hemel Hempstead, UK).

UV measurements were carried out with a Jasco V-360 Spectrophotometer (Jasco International, Tokyo, Japan).

HPLC-MS was performed with a Thermo Finnigan Surveyor LC pump equipped with a Thermo Finnigan Surveyor photodiode array detector and interfaced with the ESI Thermo Finnigan LCQ Advantage spectrometer using column and elution parameters reported previously.

### 2.2 The mutant lid-loop *B. subtilis* GGT (LL-BsGGT)

The mutant enzyme LL-BsGGT was obtained during our studies about the role of the lid-loop in GGTs.^60^

### 2.3 Enzyme activity assay

Enzyme activities were measured for BsGGT (0.639 mg mL^−1^) and LL-BsGGT (0.524 mg mL^−1^) prior to every experiments, following the procedure reported for *B. subtilis* (natto),^55^ with some modifications.

Reactions (2.0 mL) were carried out in plastic cuvettes containing 1 mM GPNA and 100 mM GlyGly in Tris buffer at pH 8.5. Reactions were initiated by adding 20 μL of diluted enzyme solution. The release of *p*-nitroaniline was monitored at 410 nm for 3 minutes at 21–23 °C, recording data every 10 s. One enzyme unit was defined as the amount of enzyme that liberates 1 μmol mL^−1^ min^−1^ of *p*-nitroaniline in the presence of glycylglycine. *p*-Nitroaniline concentrations were estimated through a calibration curve.

### 2.4 Assessment of hydrolase and transpeptidase activities

Hydrolase and transpeptidase activities were evaluated with the same procedure used for enzyme activity assays, in the presence and in the absence of GlyGly as the acceptor, respectively. Experiments were performed in duplicate using unstandardized solutions of the three enzymes (BsGGT, EcGGT, LL-BsGGT).

### 2.5 pH-activity profile of LL-BsGGT

Measurements were carried out in the same conditions of enzyme activity assays, in the presence (“transpeptidation”) and in absence (“hydrolysis”) of added GlyGly (100 mM), using buffers at different pH values from 7 to 10. In particular, pHs 7.0, 7.5, 8.0, 8.5 were controlled by using 0.1 M Tris HCl buffers, while pHs 9.0, 9.5, 10.0 through 0.1 M sodium carbonate/sodium hydrogencarbonate buffers. Experiments were performed in duplicate.

### 2.6 Substrate specificity

Measurements were carried out in the same conditions of enzyme activity assays, by replacing GlyGly with the proper acceptor (see Table 1) at 100 mM concentration. Experiments were performed in triplicate, using the wt BsGGT and the mutant enzyme LL-BsGGT after normalization of their activity in the absence of added acceptor.

**Table 1:** Acceptor substrate specificity of LL-BsGGT^a^

Entry	Acceptor	Relative activity
1	Gly-Gly	100
2	None	3.91 ± 1.01 A
3	l-Ala	2.27 ± 0.18
4	l-Cys	0.82 ± 1.01
5	l-His	19.41 ± 2.84 C
6	l-Ile	7.45 ± 2.83 B
7	l-Leu	8.98 ± 0.08 B
8	l-Met	21.95 ± 2.96 C
9	d-Met	8.17 ± 0.35 B
10	l-Phe	8.59 ± 0.45 B
11	l-Ser	4.03 ± 0.69 A
12	l-Val	4.54 ± 1.55 A
13	*S*-Methyl-l-Cys	16.43 ± 0.83 C
14	*S*-Ethyl-l-Cys	19.16 ± 0.55 C
15	*S*-Allyl-l-Cys	46.31 ± 5.15
16	Ala-Gly	65.43 ± 3.33
17	Abu-Gly	38.23 ± 2.32
18	Val-Gly	10.32 ± 1.65 B
19	Leu-Gly	4.44 ± 0.57 A

a Experiments were carried out at pH 8.5 in Tris HCl buffer using 1 mM γ-glutamyl-*p*-nitroanilide as the donor substrate in the presence of 100 mM acceptors in order to favor transpeptidation over hydrolysis and autotranspeptidation reactions. Liberation of *p*-nitroaniline was monitored continuously at 410 nm and initial velocities were derived from the slopes of the resulting curves in their initial, linear range. Results are expressed in relation to the activity measured using GlyGly as the acceptor, taken as 100. Values below that recorded in the absence of the acceptor substrate are assumed as a lack of transpeptidase activity. Values labelled with the same letter are not statistically different at 95% confidence level (Tukey test, *p* > 0.05).

### 2.7 Pre-column derivatization procedure

Standard solutions for calibration curves or aliquots of reaction mixtures to be analyzed (20 μL) were first diluted 1 : 20 with water. 100 μL of the diluted solution was transferred into a pyrex tube equipped with a perforated screw cap fitted with a forcible sealing septum. 50 μL of 5 mM l-serine in water was added as the internal standard, followed by 350 μL borate buffer at pH 8.5. The mixture was shaken and 500 μL of 10 mM 1-fluoro-2,4-dinitrobenzene (Sanger's reagent) solution in acetone was added. The sealed tube was heated at 70 °C for 45 minutes in the dark. Then a needle was introduced into the septum and the tube was left in the heated water bath for further 10 minutes, to evaporate most of acetone. The tube was cooled under running water and 300 μL of the resulting mixture was diluted 1 : 2 with 0.1% TFA solution, affording the sample for HPLC analysis.

### 2.8 LL-BsGGT-catalyzed reactions

#### 2.8.1 Assessment of reaction conditions

Reaction conditions (effect and enzyme concentration and pH) were preliminary investigated as described in 2.3 (enzyme activity assay) and in 2.5 (pH-activity profile of LL-BsGGT), respectively, using ValGly or *S*-allylcysteine as acceptor substrates (100 mM) instead of GlyGly.

Effect of temperature was investigated by carrying out reactions at analytical level at different temperatures, as described in 2.8.2.

#### 2.8.2 Analytical level. General procedure

A 1 mL-mixture of glutamine and the acceptor substrate (valylglycine or *S*-allylcysteine), each at 100 mM concentration in 0.1 M Tris·HCl buffer at the right pH (8.5 for ValGly, 9.5 for *S*-allylcysteine) and LL-BsGGT (0.6, 1.2 or 4.0 U mL^−1^) was stirred in a thermostatic water bath at a fixed temperature (either 25 or 40 °C). At fixed time points, 20 μL aliquots were withdrawn, derivatized as described previously and analyzed by HPLC.

#### 2.8.3 Effect of a further addition of the donor substrate

A 1 mL-mixture of glutamine (100 mM), valylglycine (100 mM) and LL-BsGGT (4.0 U mL^−1^) in 0.1 M Tris·HCl buffer at pH 8.5 was stirred in a thermostatic water bath at 25 °C. After 45 min reaction time, a further equivalent of glutamine (14 mg) was added. Reaction was monitored at fixed time points as described in 2.8.2.

#### 2.8.4 Preparative level. General procedure

A 5 mL-mixture of glutamine and the acceptor substrate (valylglycine or *S*-allylcysteine), each at 100 mM concentration in 0.1 M Tris·HCl buffer at the right pH (8.5 for valylglycine, 9.5 for *S*-allylcysteine) and LL-BsGGT (0.6 or 4.0 U mL^−1^) was stirred in a thermostatic water bath at 25 °C for the appropriate time (5 hours for valylglycine and 6 hours for *S*-allylcysteine). Reactions were stopped by inactivating the enzyme at 95 °C for 10 min. The pH of the mixture was adjusted to 9.5 with 1 M NaOH and the solution was loaded onto a column packed with Dowex 1 × 8 ion exchange resin in the acetate form. The column was eluted with water (4 column volumes) and then with a stepwise gradient of acetic acid solution (0.5, 1.0, 1.5, and 2.0 M, three column volumes each), collecting eluate in fractions. On the basis of TLC analysis (silica gel, *n*-BuOH/water/AcOH 3 : 1 : 1; staining reagent: ninhydrin), fractions were combined and freeze-dried.

#### 2.8.5 γ-Glutamyl-l-valylglycine

Obtained in 35% isolated yield after 5 hours reaction time using 0.6 U mL^−1^ LL-BsGGT.

^1^H-NMR (400 MHz; D_2_O): *δ* 4.17 (d, *J* = 6.8 Hz, 1H, Hα Val), 4.00 (d, *J* = 17.8 Hz, 1H, Hα1 Gly), 3.97 (d, *J* = 17.8 Hz, 1H, Hα2 Gly), 3.83 (t, *J* = 6.4 Hz, 1H, Hα Glu), 2.61–2.48 (m, 2H, Hγ Glu), 2.19–2.09 (m, 3H, Hβ Glu and Hβ Val), 0.97 (d, *J* = 6.8 Hz, 3H, Me1 Val), 0.98 (d, *J* = 6.8 Hz, 3H, Me2 Val).

^13^C NMR (101 MHz; D_2_O): *δ* 174.9 (COγ Glu), 174.1 (CO Val), 173.49 (CO Gly), 173.40 (COα Glu), 59.6 (Cα Val), 53.8 (Cα Glu), 41.3 (Cα Gly), 31.1 (Cγ Glu), 29.9 (Cβ Val), 26.1 (Cβ Glu), 18.3 (Me-1 Val), 17.3 (Me-2 Val).

ESI-MS, negative mode: *m*/*z* 302.56 [M − H]^−^; 324.55 [M − 2H + Na]^−^

#### 2.8.6 γ-Glutamyl-*S*-allyl-l-cysteine

Obtained in 21% isolated yield after 6 hours reaction time using 0.6 U mL^−1^ LL-BsGGT.

^1^H-NMR (400 MHz; D_2_O): *δ* 5.84 (ddt, *J* = 17.1, 9.9, 7.2 Hz, 1H, vinyl CH), 5.23–5.17 (m, 2H, vinyl CH_2_), 4.56 (dd, *J* = 8.2, 4.8 Hz, 1H, Hα Cys), 3.90 (t, *J* = 6.3 Hz, 1H, Hα Glu), 3.23 (d, *J* = 7.2 Hz, 2H, allyl CH_2_), 3.05 (dd, *J* = 14.1, 4.8 Hz, 1H, Hβ1 Cys), 2.89 (dd, *J* = 14.1, 8.2 Hz, 1H, Hβ2 Cys), 2.61–2.49 (m, 2H, Hγ Glu), 2.27–2.13 (m, 2H, Hβ Glu).

^13^C NMR: see ref. 67.

ESI-MS, negative mode: *m*/*z* 289.15 [M − H]^−^; 311.16 [M − 2H + Na]^−^.

## 3. Results and discussion

### 3.1 Transpeptidase activity of LL-BsGGT

**Fig. 3:**
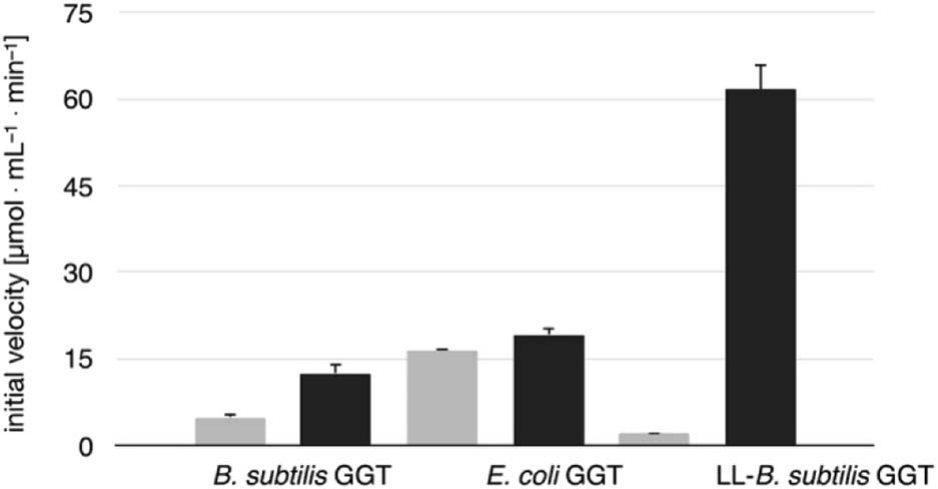
Comparison of the activities of BsGGT, EcGGT and LL-BsGGT in the absence (grey bars) and in the presence (black bars) of the added acceptor GlyGly. Measurements were carried out at pH 8.5 in Tris HCl buffer, by continuously monitoring the release of *p*-nitroaniline at 410 nm and deriving the initial reaction rates from the slope of the resulting curves in their linear range. Results are averages of three experiments; error bars refer to standard deviation.

The transpeptidase activity of the mutant LL-BsGGT was preliminarily compared to those of the wt enzymes EcGGT and BsGGT. In the standard activity assay, based on the use of the chromogenic γ-glutamyl-*p*-nitroanilide as the donor substrate and glycylglycine as the standard acceptor, the activity of LL-BsGGT was much higher (*ca.* 23 folds) in the presence of added GlyGly than in its absence (Fig. 3). The result is quite remarkable, in that the increase in the enzymatic activity of bacterial GGTs due to the presence of an added acceptor is normally modest,^52,54,61^ and the mutant LL-BsGGT differs from the parent wt BsGGT only for the presence of the inserted lid-loop, thus underlining the importance of this structural feature in modulating enzyme functions.

The activity of LL-BsGGT was then compared with that of the wt counterpart after normalization of the two enzymes through reactions carried out in the absence of the acceptor substrate. In repeated experiments the activity of the wt enzyme was 2.5–4.6-fold higher in the presence of GlyGly than in its absence, while the mutant LL-BsGGT showed an unusual 25–30-fold increase in activity in the presence of GlyGly. Overall, the mutant enzyme appeared to be *ca.* 5–7-fold more effective in catalyzing the transpeptidation reaction than the wt. In the absence of the acceptor substrate, the measurement of specific activities of the two enzymes gave quite similar results, namely 1122 and 1304 μmol min^−1^ mg^−1^ for the wt and the mutant enzyme, respectively, while in the presence of GlyGly the activities were 5198 and 29 686 μmol min^−1^ mg^−1^, respectively.

### 3.2 pH dependence

**Fig. 4:**
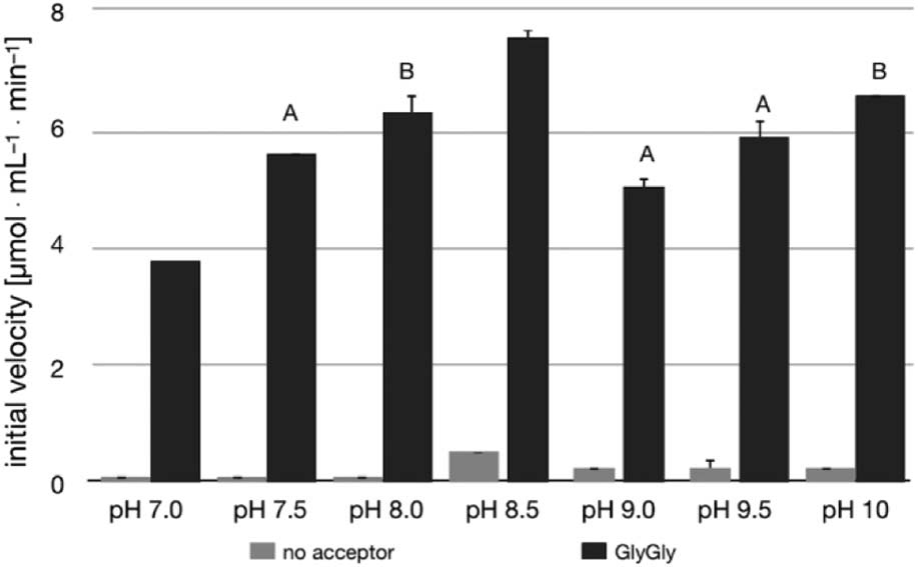
pH-activity profile for LL-BsGGT in the absence (grey bars) and in the presence (black bars) of GlyGly as the acceptor substrate. Measurements at pH 7.0–8.5 were carried out in Tris HCl buffer; measurements at pH 9.0–10 were carried out in sodium hydrogen carbonate/sodium carbonate buffer. Bars labelled with the same letter are not statistically different at 95% confidence level (Tukey test, *p* > 0.05).

The pH-activity profile of LL-BsGGT in the absence of the acceptor substrate seems to assume a flat bell shape, while in the presence of GlyGly the activity is favored by basic pH values (Fig. 4) according to the assumption that pH affects not only the intrinsic enzymatic activity, but also the proportion of the acceptor substrate present in its non-protonated, nucleophilic form.^51,52^ As a consequence, the optimum pH for transpeptidase activity cannot be univocally determined, as it depends on the p*K*_a_ of the nucleophilic amino group of the acceptor substrate.

### 3.3 Transpeptidase activity of LL-BsGGT towards selected acceptor substrates

**Fig. 5:**
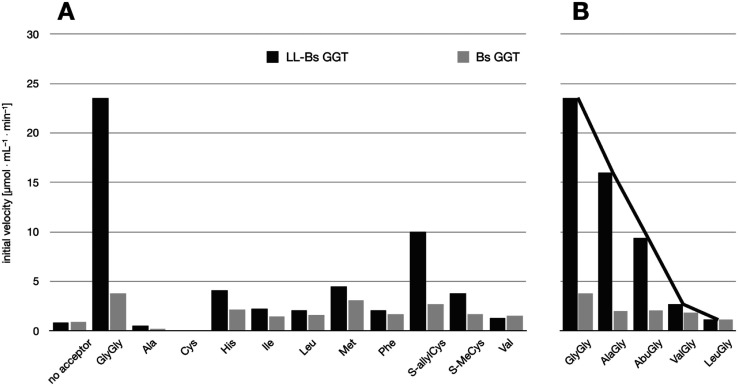
Comparison of the activities of BsuGGT and LL-BsuGGT towards selected acceptors. The activities of the two enzymes were prior normalized in the reaction without added acceptor. (A) Activity towards simple amino acids. (B) Activity towards some representative aminoacylglycines with increasing steric demand near the nucleophilic nitrogen atom (see Fig. 6).

The relative activities towards different acceptor substrates measured for LL-BsGGT in relation to that of GlyGly are reported in Table 1. Fig. 5A shows the activities measured for selected acceptors in comparison with the activities displayed by the wt counterpart, after normalization of enzyme activities as reported above.

In addition to available data,^56^ also *S*-substituted cysteines *S*-methyl- and *S*-allylcysteine proved to be good acceptors for the wt enzyme. On the contrary, the transpeptidation of alanine and cysteine were negligible, and comparable to the mock reactions in the absence of the acceptor substrate. A possible inhibitory activity of alanine towards BsGGT has been previously hypothesized.^52^

The mutant LL-BsGGT generally gave better results with respect to the wt enzyme, although the improvement was modest for most of the tested acceptors. The mutant enzyme showed low activities when apolar aromatic and branched-chain amino acids were used as acceptor substrates (Table 1, entries 6, 7, 10 and 12), with a *ca.* 1.5-fold higher activity with respect to the wt counterpart (Fig. 5A). A modest improvement was observed also for methionine (Table 1, entry 8), which is among the best acceptors for BsGGT. Conversely, the *S*-substituted cysteines showed the higher activities (Table 1, entries 13–15), with a 2.5–4.1 increase in comparison with the wt enzyme (Fig. 5A).

**Fig. 6:**
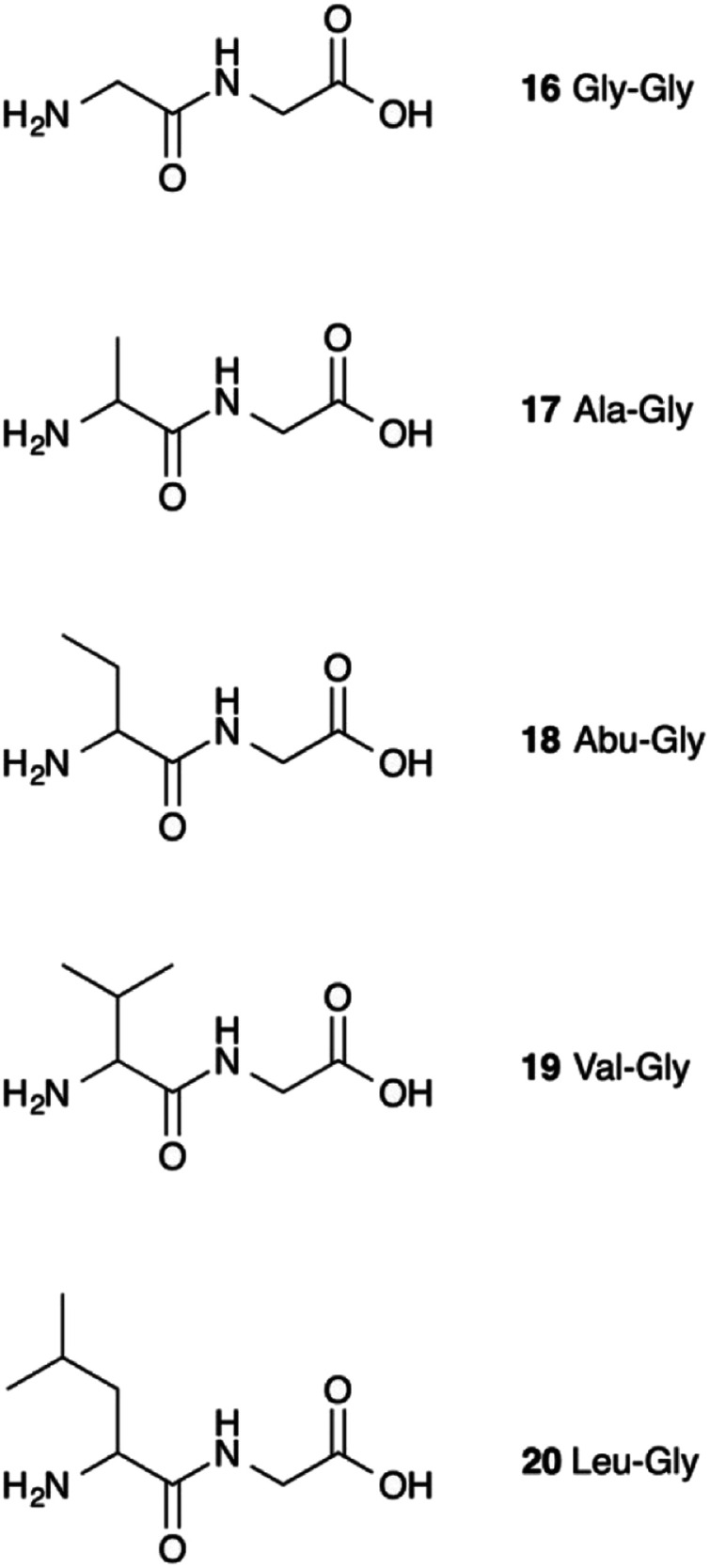
Acceptor aminoacyl-glycines with increasing steric demand near the nucleophilic amino group.

It therefore appears that LL-BsGGT showed an astonishingly high activity only towards GlyGly as the acceptor. The result is intriguing, as the active sites of the wt and mutant enzymes are essentially the same, the main difference being the presence of the lid-loop covering the γ-glutamyl binding site on the mutant. As the lid-loop has a gate function, allowing the selection of the donor substrate on the basis of steric requirements,^60^ the mutant LL-BsGGT may have become more selective also in the recognition of the acceptor substrate. In order to test this hypothesis, aminoacyl derivatives of glycine with increasing steric demand (16–20, Fig. 6) were assayed (Table 1, entries 1 and 16–19). While the transpeptidase activity of wt BsGGT towards compounds 16–20 varied comparatively little (Fig. 5B), the mutant enzyme seemed much more selective in recognizing the acceptor substrate on the basis of the steric bulkiness. Indeed, the activities measured in LL-BsGGT-catalyzed reactions decreased as the steric demand of the amino acid side chains increased.

Curiously enough, in several independent experiments the activities showed always an inverse linear correlation with the number of carbon atoms (0 for GlyGly, 3 for Val) on the acceptor side chains (Fig. 5B). The activity of the mutant enzyme towards LeuGly 20 was only slightly higher than that in the absence of acceptor substrate (Table 1, entry 19). The relatively high activities observed in reactions involving the sulfur-containing amino acids (Table 1, entries 8 and 13–15) could then be related to the minor steric requirements of their straight side-chains with respect to the more demanding ones of branched-chain and aromatic amino acids.

Unfortunately, while the γ-glutamyl binding site of several GGTs has been investigated in detail through X-ray crystallography,^59,62–66^ the acceptor binding site remains, on the contrary, elusive. It is therefore difficult to correlate the hydrolase and transpeptidase activities of GGTs towards different acceptor substrates on structural basis. Computational studies are ongoing in our laboratories in order to gain insights into this topic.

### 3.4. Synthetic application of mutant LL-BsGGT

The possible use of the mutant LL-BsGGT as biocatalyst for the synthesis of γ-glutamyl derivatives of applicative interest was tested towards both a dipeptide and an amino acid. The acceptor dipeptide was valylglycine 19 and the amino acid was *S*-allyl cysteine. The choice of the two acceptors was dictated by our interest in the search of an enzyme-catalyzed reaction able to afford taste-active compounds with kokumi properties.^7,35^

#### 3.4.1 Enzymatic synthesis of γ-glutamylvalylglycine

The dipeptide valylglycine 19 was prepared through the classical methods of peptide synthesis in solution (see ESI†).

**Fig. 7:**
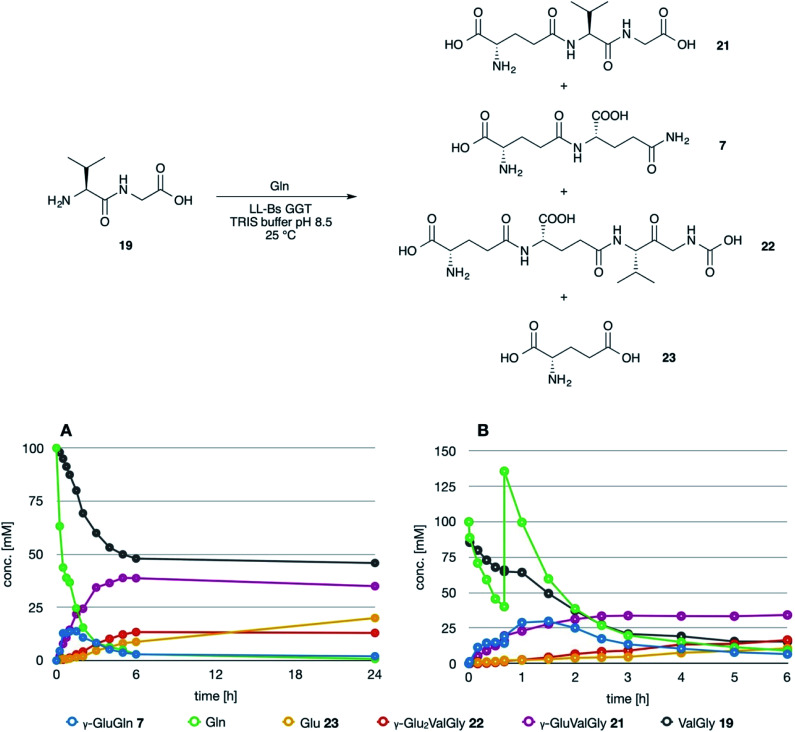
Time course of the LL-BsGGT-catalyzed synthesis of γ-GluValGly. (A) Reaction carried out using equimolar amounts of donor and acceptor substrates. (B) Reaction carried out by supplementing one equivalent of the donor substrate after 45 min reaction time (see text for details).

**Fig. 8:**
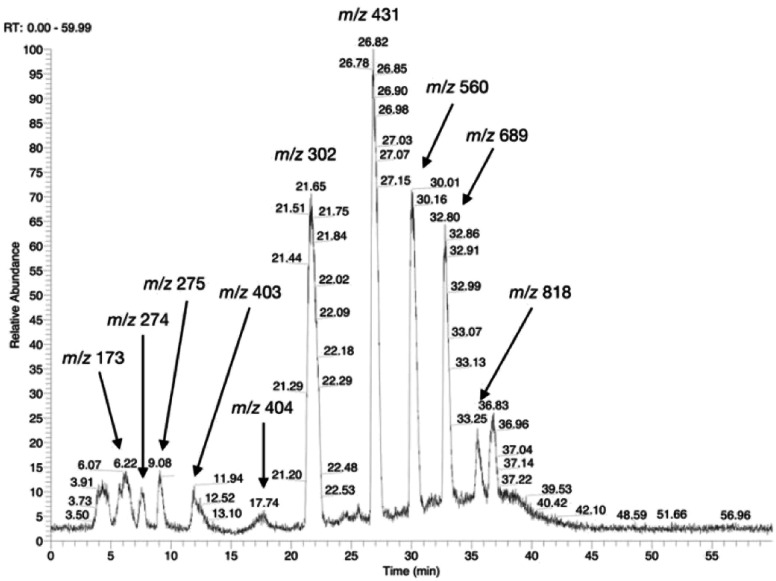
HPLC-MS chromatogram of an underivatized sample from the LL-BsGGT-catalyzed reaction between Gln as the donor and ValGly as the acceptor at the end of the reaction. Detection was in negative mode. Recorded *m*/*z* values are reported and are attributable as follow: *m*/*z* 173: ValGly; *m*/*z* 274: γ-GluGln; *m*/*z* 275: γ-GluGlu; *m*/*z* 403: γ-Glu_2_Gln; *m*/*z* 404: γ-Glu_2_Glu; *m*/*z* 302: γ-GluValGly; *m*/*z* 431: γ-Glu_2_ValGly; *m*/*z* 560: γ-Glu_3_ValGly; *m*/*z* 689: γ-Glu_4_ValGly; *m*/*z* 818: γ-Glu_5_ValGly.

It is to note that, although the activity of LL-BsGGT towards Val-Gly as acceptor compound seemed to be rather low (Table 1, entry 18), it was still 1.5 fold higher than that of the wt enzyme (Fig. 5A). Enzymatic activity in the presence of Val-Gly varied little with pH between 7 and 10, with higher yields obtained at pH 8.5. The reaction was first tested at 25 °C in the presence of equimolar amounts of glutamine as the donor substrate and Val-Gly at 100 mM and was monitored by HPLC upon pre-column derivatization with 1-fluoro-2,4-dinitrobenzene (Sanger's reagent) (Fig. 7A). The concentration of the donor substrate glutamine dropped rapidly in addition to the desired γ-GluValGly 21, also γ-GluGln 7 and γ-Glu_2_Gln^60^ were identified. Notably, the concentration of γ-GluGln 7 rose quickly at the beginning of the reaction, indicating a strong autotranspeptidase activity of the enzyme, and then decreased slowly, while the concentration of the transpeptidation product 21 reached a plateau at 4–5 hours, and then remained fairly constant up to 24 hours. The concentration of the hydrolysis product glutamic acid 23 rose slowly in the initial stages, and then increased when the concentrations of glutamine and γ-GluGln 7 fell below a lower threshold. HPLC-MS analysis of the non-derivatized reaction mixture (Fig. 8) showed the presence of numerous poly-γ-glutamylated compounds, deriving from both the donor glutamine and the acceptor ValGly (*e.g.*22 in Fig. 7). This behavior was not unexpected, as the enzyme can accept as substrates the γ-glutamyl derivatives formed in the early stages of the reaction. Thus, γ-GluGln 7 is formed as long as Gln is present in sufficient amount and behaves as a donor substrate when the concentration of glutamine drops. Also the transpeptidation product γ-GluValGly is recognized as a donor substrate, but the presence of ValGly ensures its continuous re-formation, maintaining its concentration fairly stable. This balance is broken by the irreversible hydrolysis reaction leading to glutamic acid, which is not recognized by GGTs as a donor substrate. However, the hydrolysis reaction is slow in comparison with transpeptidation, so explaining the slow decline of the product from six to 24 hours, paralleled by the equally slow increase of glutamic acid concentration. Maximum concentration of the desired γ-GluValGly was 38 mM and was reached between four and six hours.

The reaction was repeated at a preparative level in a volume of 5 mL and after 5 hours reaction time the product was obtained in 35% isolated yield after ion exchange column chromatography. This amount is in good agreement with the concentration of the product in the reaction mixture as determined by HPLC, thus confirming the reliability of the analytical method used for reaction monitoring.

The ability of the synthetized γ-glutamyl derivatives to also act as acceptor substrates affording poly-γ-glutamylated compounds, represents an undesirable trait of the reaction. This functional feature of the mutant enzyme is very likely an inheritance from the parent wt BsGGT. Indeed, it has been shown that BsGGT is able to accept polymeric substrates, being actually a poly-γ-glutamic acid (γ-PGA) hydrolase.^52,60,68^ γ-PGA is a natural polymer composed by very long chains of l- and d-glutamic acid residues in different proportions, linked together through amide bonds formed by the γ-carboxyl group of a glutamic acid residue and the amino group of the next residue in the chain.^69^ It is produced by several microorganisms and notably by some *Bacillus* spp.^70^

For the principle of microscopic reversibility, BsGGT should then be able to catalyze the formation of oligomers under appropriate conditions.

Attempts to improve the conversion of the substrates into the desired product by raising the concentration of the enzyme in the reaction medium or the temperature up to 40 °C affected only the reaction time, but did not result in a substantial improvement in product formation or changes in products distribution. The observation that the concentration of γ-GluValGly remained quite stable in the reaction mixture after the disappearance of the donor substrates prompted us to try a fed-batch procedure, restoring the concentration of the donor glutamine immediately before the concentration of the transpeptidation product reached the plateau (Fig. 7B). A higher amount of enzyme was used in these experiments, so as to shorten the reaction time. After the addition of the second equivalent of glutamine, the concentration of γ-GluGln 7 showed an initial steep increase; when its concentration started to decrease, the concentration of γ-Glu-γ-GluValGly grew more significantly than that of γ-GluValGly (Fig. 7B). At the same time, several peaks of decreasing intensities, attributable to poly-γ-glutamyl glutamine derivatives (at least 5 γ-glutamyl residues linked to a single glutamine moiety), eluted before γ-Glu_2_Gln. Also, poly-γ-glutamylated derivatives of the acceptor valylglycine were identified. This result suggests that the transpeptidase activity of the enzyme is affected at least in part by the relative concentration of the possible acceptor species present in the reaction mixture. When the concentration of the first transpeptidation product, γ-GluValGly, nearly equaled the concentration of ValGly, it started to compete to a significant extent with the latter as the acceptor substrate. This might explain the steady concentration of the first product from a certain time point on, usually 4–6 h, and the appearance of several peaks attributable to poly-glutamylated species in the HPLC-MS spectra, the concentration of which decreased with the increase in number of bound γ-glutamyl residues.

#### 3.4.2 Enzymatic synthesis of γ-glutamyl-*S*-allylcysteine

The reaction in which *S*-allylcysteine was used as the acceptor substrate showed a similar trend. The reaction was carried out at an analytical level at pH 9.5 and 25 °C, and HPLC monitoring revealed that a maximum product concentration of *ca.* 22 mM was obtained between 5 and 6 hours. At a preparative level, the reaction allowed the isolation of the product 9 in 21% yield, confirming also in this case the good correlation with the concentrations estimated by HPLC. Surprisingly enough, the yield of γ-glutamyl-*S*-allylcysteine 9 was lower than that of γ-glutamylvalylglycine obtained in the previous reaction, despite the activity of the enzyme was higher in the presence of the former than in the presence of the latter (Table 1 and Fig. 5). A possible explanation could rely on the higher activity of the enzyme not only towards *S*-allylcysteine, but also towards the γ-glutamyl derivatives thereof. The product did not accumulate in the reaction mixture because it was used as acceptor in further γ-glutamyl-transfer reactions. Unfortunately, except the peak attributable to γ-glutamyl-γ-glutamyl-*S*-allylcysteine, which was easily assigned, it was not possible to identify and quantify other peaks attributable to poly-γ-glutamylated-*S*-allylcysteine derivatives, as those would be expected in a crowded area of the chromatogram. It is also conceivable that poly-γ-glutamylated species could compete as acceptor substrates being structurally similar to γ-PGA, the physiological substrate of the wt enzyme, *i.e.* the glutamate moiety of the molecule, and not the *S*-allylcysteine portion, was recognized by the enzyme.

In summary, our results confirm that the mutant enzyme inherited some functional features from the wt BsGGT. BsGGT is indeed considered a hydrolase with adventitious transpeptidase activity and relaxed acceptor substrate specificity.^56,67,71^ In addition, present results suggest that the transpeptidase activity of the mutant enzyme depends in part on the concentration of the possible nucleophilic species in the reaction mixture.

## 4. Conclusions

In our ongoing search on the possible exploitation of bacterial GGTs as biocatalysts, the transpeptidase activity of a mutant GGT, obtained by inserting the lid-loop from *E. coli* GGT on the structure of *B. subtilis* GGT, naturally lacking it, has been investigated.

Our results suggest that the lid-loop is able to shield the γ-glutamyl enzyme intermediate from bulk solvent, preventing hydrolysis and thus favoring the transpeptidation reaction. In addition, it contributes to substrate selection on the basis of steric requirements near the nucleophilic amino group. On the other hand, being the active site of the mutant enzyme essentially identical to that of the wt, the mutant enzyme catalyzes the formation of poly-γ-glutamylated species, thus reflecting the ability of the parent *B. subtilis* GGT to accept polymeric substrates. The substrate specificity appears to be relaxed and the relative concentration of the possible nucleophiles in the reaction mixture gain importance in determining products distribution.

The mutant enzyme does appear promising as a biocatalyst for preparative purposes in case poly-glutamylated species are the desired products, however these studies allow a deeper understanding of GGTs behavior. Another interesting aspect emerged from our investigation: as previously reported,^52^ there does not appear to be a straightforward relationship between the enzymatic activity towards a given acceptor substrate, as measured through the standard colorimetric assay, and the ability of the same acceptor to afford satisfactory results in reactions carried out at a preparative level. Indeed, the actual event monitored by the colorimetric assay is the release of *p*-nitroaniline due to the formation of the γ-glutamyl-enzyme intermediate. Then the γ-glutamyl-enzyme intermediate must be resolved and, using a large excess of acceptor substrate, the transpeptidation reaction is assumed to be the preferred pathway. However, such a large excess of acceptor substrate cannot be replicated at a preparative level, so other reaction pathways, such as hydrolysis, autotranspeptidation and the formation of poly-glutamylated species, become evident. Indeed, the assay based on the use of γ-glutamyl-*p*-nitroanilide was assessed and validated for applications with mammalian GGTs^72^ and it is thus conceivable that the same assay is not able to account satisfactorily for the different behavior exerted by bacterial GGTs in standard reaction conditions.

## Conflicts of interest

Authors have no conflicts of interest to declare.

## Supplementary Material

RA-009-C9RA05941E-s001
