# Supplementary material for: Effect of the inserted active-site-covering lid loop on the catalytic activity of a mutant B. subtilis γ-glutamyltransferase (GGT)
Source: RSC Adv. 2019 Oct 28;9(60):34699–709. doi: 10.1039/c9ra05941e (PMC9073855; doi:10.1039/c9ra05941e)
Supplement: RA-009-C9RA05941E-s001 [file RA-009-C9RA05941E-s001.pdf]

Electronic Supplementary Material (ESI) for RSC Advances.
This journal is © The Royal Society of Chemistry 2019

Effect of the inserted active site-covering lid loop on the catalytic activity of a mutant *B. subtilis* GGT

Michela Massone,[a] Cinzia Calvio,[b] Marco Rabuffetti,[c] Giovanna Speranza[a,d] and Carlo F. Morelli*[a]

[a] Department of Chemistry, Università degli Studi di Milano, via Golgi, 19 - 20133 Milano - Italy
[b] Department of Biology and Biotechnology, Università degli Studi di Pavia, via Ferrata,1 - 27100 Pavia - Italy
[c] Dipartimento di Scienze per gli Alimenti, la Nutrizione e l'Ambiente, Università degli Studi di Milano, via Mangiagalli, 25 - 20133 Milano - Italy
[d] Istituto di Scienze e Tecnologie Molecolari (INSTM, CNR, ℅ Department of Chemistry, via Golgi, 19 - 20133 Milano - Italy

Supporting Information

Synthesis of L-valylglycine and γ-glutamyl-L-valylglycine

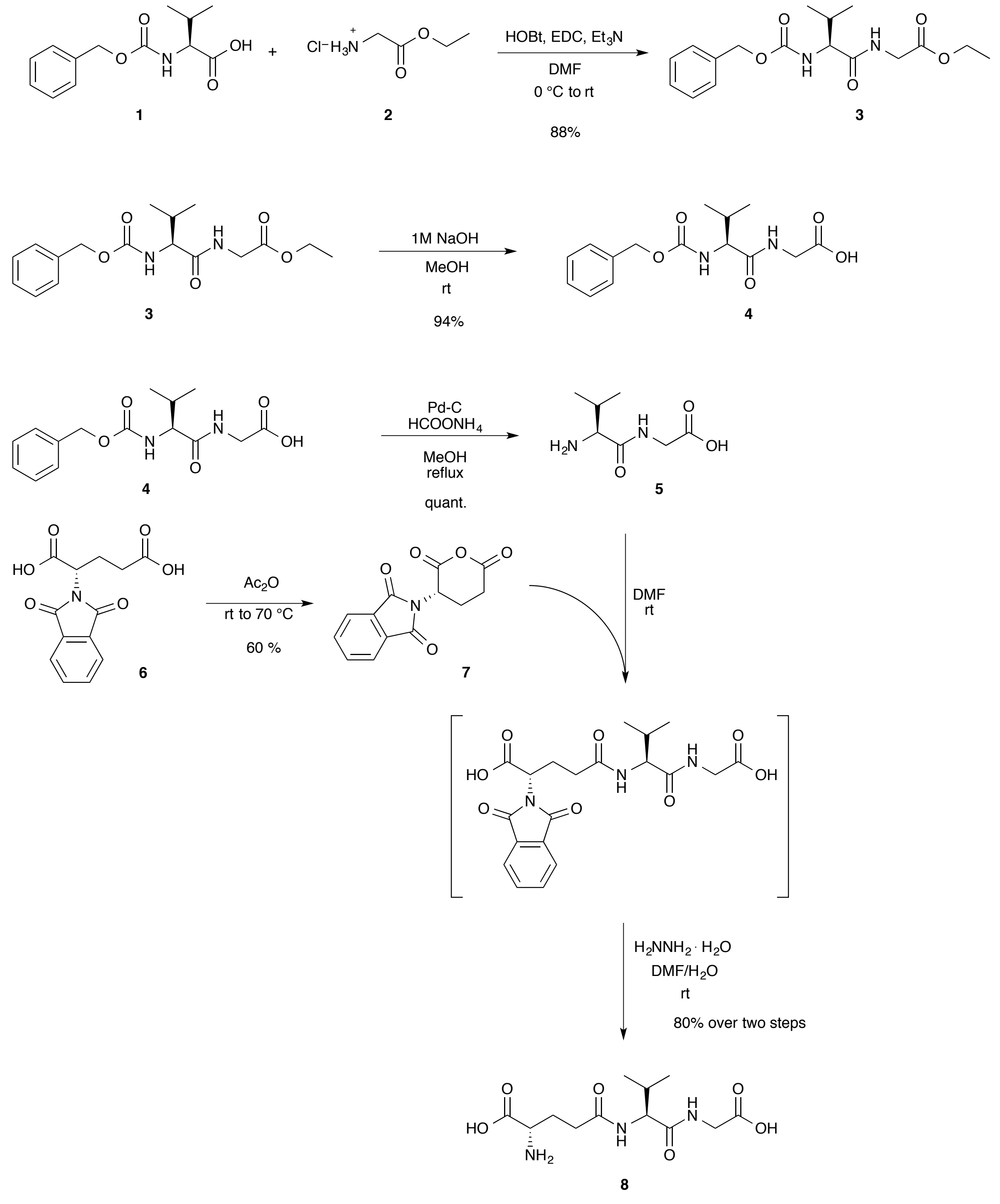

Scheme S1. Synthesis of L-valylglycine (**5**) and γ-glutamyl-L-valylglycine (**8**).

General

L-valine, glycine ethyl ester hydrochloride, *N*-phtaloyl-L-glutamic acid, acetic anhydride, benzyl chloroformate, 1-hydroxybenzotriazole (HOBt), 1-ethyl-3-(3-dimethylaminopropyl)carbodiimide (EDC) and dry DMF were from Sigma-Aldrich and were used as received.

Solvents were distilled by standard methods prior to use.

N-benzyloxycarbonyl-L-valine (**1**) was prepared by standard procedure.

N-phtaloyl-L-glutamic acid anhydride (**7**) was prepared from *N*-phtaloyl-L-glutamic acid (**6**) as described previously.[1]

Analytical TLC was performed on silica gel F_{254} pre-coated aluminum sheets (0.2 mm layer) (Merck, Darmstadt, Germany). Elution solvents are reported in the paragraph of the specific product's preparation. Detection: UV lamp (λ 254 nm), 4.5% w/v $CeSO_4/(NH_4)_6Mo_7O_{24}\cdot 4H_2O$ solution or 5% w/v ninhydrin solution in ethanol, followed by heating at 150 °C ca.

Ion exchange column chromatography was performed by using Dowex 1x8 resin 200-400 mesh (Aldrich, Darmstadt, Germany) in the acetate form. Resin was equilibrated before use with 5 volumes of 0.5 M acetic acid.

^{1}H-NMR and ^{13}C-NMR spectra were acquired at 400.13 MHz and 100.61 MHz, respectively, on a Bruker Advance 400 spectrometer (Bruker, Karlsruhe, Germany) interfaced with a workstation running Windows operating system and equipped with a TOPSPIN software package. ^{13}C signal multiplicities were based on attached proton test experiments (APT). Chemical shifts are given in ppm (δ) and are referenced to solvent signal (δ_H $CDCl_3$ 7.26; δ_C $CDCl_3$ 77.16; δ_H DMSO 2.50; δ_C DMSO 39.52; δ_H D_2O 4.79 ppm) or to TSP (3-(trimethylsilyl)propionic-2,2,3,3-d4 acid sodium salt) as external standard (δ_{Me} 0.00 ppm).

Spectra analyses were carried out using inmr Reader software (www.inmr.net, last access May, 2019).

ESI-MS spectra were recorded on a Thermo Finnigan LCQ Advantage spectrometer (Hemel Hempstead, UK).

Synthesis of L-valylglycine **5**

To a cooled (0 °C) solution of *N*-benzyloxycarbonyl-L-valine (**1**, 6.93 g, 27.5 mmol), glycine ethyl ester hydrochloride (**2**, 3.84 g, 27.5 mmol), HOBt (5.07 g, 37.5 mmol) and EDC (7.17 g, 37.5 mmol) in dry DMF (60 mL), triethylamine (17.5 mL, 125 mmol) was added dropwise over 15 min. The reaction mixture was stirred for 24 h, allowing the temperature to reach room temperature. After completion of the reaction (TLC monitoring, silica gel, DCM/MeOH/formic acid 9:1:0.5) the mixture was cooled to 0 °C and water (60 mL) was slowly added dropwise under vigorous stirring. The formed precipitate was collected by filtration and dried under reduced pressure over KOH. White amorphous solid, 8.20 g, 88.6% yield.

N-benzyloxycarbonyl-L-valylglycine ethyl ester (**3**)

^{1}H NMR (400 MHz; $CDCl_3$): δ 7.38-7.29 (m, 5H), 6.36 (t, *J* = 4.3 Hz, 1H), 5.32 (d, *J* = 7.9 Hz, 1H), 5.12 (d, *J* = 12.4 Hz, 1H), 5.10 (d, *J* = 12.3 Hz, 1H), 4.21 (q, *J* = 7.1 Hz, 2H), 4.10-3.96 (m, 3H), 2.18 (8, *J* = 6.6 Hz, 1H), 1.28 (t, *J* = 7.1 Hz, 3H), 0.99 (d, *J* = 6.8 Hz, 3H), 0.94 (d, *J* = 6.8 Hz, 3H).
^{13}C NMR (101 MHz; $CDCl_3$): δ 171.6, 169.7, 156.6, 136.3, 128.7, 128.37, 128.23, 67.3, 61.8, 60.5, 41.4, 31.2, 19.4, 17.8, 14.3.
ESI-MS (positive mode): *m/z* 359.41[M + Na]$^+$

N-benzyloxycarbonyl-L-valylglycine ethyl ester (**3**, 5.00 g, 14.8 mmol) obtained as described was dissolved in MeOH (150 mL) and 1M NaOH (18 mL) was added dropwise over 10 min. The mixture was stirred at room temperature for 4 h (TLC monitoring, silica gel, AcOEt/hexane 1:1); then the methanol was evaporated under reduced pressure. The oily residue was taken up in water (80 mL) and the aqueous phase was washed with AcOEt (2 x 50 mL), acidified with 2M HCl up to pH ca 2 and extracted with AcOEt (3 x 60 mL). The combined organic layers were washed with saturated NaCl (100 mL), dried over sodium sulfate and evaporated under reduced pressure. White, amorphous solid, 4.28 g (93.8% yield).

N-benzyloxycarbonyl-L-valylglycine (**4**)

^{1}H NMR (400 MHz; DMSO-d_6): δ 12.53 (s, 1H), 8.21 (t, *J* = 5.7 Hz, 1H), 7.37-7.26 (m, 5H), 5.04 (d, *J* = 12.6 Hz, 1H), 5.01 (d, *J* = 12.7 Hz, 1H), 3.89 (dd, *J* = 8.9, 7.0 Hz, 1H), 3.80 (dd, *J* = 17.5, 5.9 Hz, 1H), 3.70 (dd, *J* = 17.5, 5.7 Hz, 1H), 1.97 (dq, *J* = 13.6, 6.8 Hz, 1H), 0.88 (d, *J* = 6.8 Hz, 3H), 0.85 (d, *J* = 6.8 Hz, 3H).
^{13}C NMR (101 MHz; DMSO-d_6): δ 171.5, 171.1, 156.1, 137.1, 128.3, 127.73, 127.63, 65.4, 60.0, 40.6, 30.3, 19.2, 18.0

To a suspension of Pd-C (10% Pd, 3.00 g) in a solution of ammonium formate (3.48 g, 55.2 mmol) in MeOH (100 mL) under Ar athmosphere, the previously obtained intermediate **4** (4.20 g, 13.6 mmol) dissolved in MeOH (60 mL) was added dropwise under stirring at room temperature over 5 min. The mixture was then gently refluxed for 1h (TLC monitoring, silica gel, DCM/MeOH/formic acid 9:1:0.5). The reaction mixture was cooled to room temperature and Pd-C was removed by filtration on a pad of celite. Evaporation of the methanol under reduced pressure afforded the desired product. White, amorphous solid, 2.36 g (99.6% yield).

L-valylglycine (**5**)

^{1}H NMR (400 MHz; D_2O): δ 3.84 (d, *J* = 17.2 Hz, 1H), 3.72 (d, *J* = 6.8 Hz, 1H), 3.63 (d, *J* = 17.2 Hz, 1H), 3.27 (s, 1H), 2.14 (8, *J* = 6.8 Hz, 1H), 0.97 (d, *J* = 6.8 Hz, 3H), 0.96 (d, *J* = 6.8 Hz, 3H).
^{13}C NMR (101 MHz; D2O): δ 176.1, 169.3, 58.9, 43.2, 29.7, 17.6, 17.0.

Synthesis of γ-glutamyl-L-valylglycine **8**

ValGly **5** (348 mg, 2.00 mmol) was suspended in dry DMF (4 mL) at room temperature under a stream of dry nitrogen. *N*-phtaloyl-L-glutamic acid anhydride (**7**, 518 mg, 2.00 mmol, prepared as reported in ref. 1) was added and the suspension was stirred for 5 h (TLC monitoring, silica gel, nBuOH/water/AcOH 4:1:1). The initial suspension became slowly a cloudy solution. Water was then added (0.4 mL), followed by hydrazine hydrate (0.6 mL, 7 mmol) under vigorous stirring. A thick precipitate formed within 2 h. Ethanol was added (2 mL) and the solid was triturated; the suspension was then stirred at 0 °C for 2 additional hours in order to favor complete precipitation. The solid was collected by filtration through a sinthered glass septum, washed in succession with cold ethanol, AcOEt and diethyl ether and dried under reduced pressure, without removing it from the septum.

The hydrazinium salt of the desired product was removed from the septum by dissolving it with small aliquots of water, leaving on the septum much of the phalylhydrazide byproduct (TLC monitoring, conditions as above). The solution was basified to pH 9.5 with 1M NaOH and charged onto a column containing a pad of Dowex 1x8 ion exchange resin in the acetate form. The resin was eluted with water until disappearance of hydrazine from the eluate (test with 2% *N*,*N*-dimethylaminobenzaldehyde in 10% aqueous HCl). The column was then eluted with a scalar gradient of acetic acid solutions (0.5, 1.0, 1.5 and 2 M, three column volumes each). Eluate was collected in fractions and checked by TLC (conditions as above). Fractions containing the reaction product were combined and freeze-dried. White, amorphous solid, 486 mg, 80% yield.

γ-glutamyl-L-valylglycine (**8**)

^{1}H NMR (400 MHz; D_2O): δ 4.17 (d, *J* = 6.8 Hz, 1H, Hα Val), 4.00 (d, *J* = 17.8 Hz, 1H, Hα1 Gly), 3.97 (d, *J* = 17.8 Hz, 1H, Hα2 Gly), 3.83 (t, *J* = 6.4 Hz, 1H, Hα Glu), 2.61-2.48 (m, 2H, Hγ Glu), 2.19-2.09 (m, 3H, Hβ Glu and Hβ Val), 0.97 (d, *J* = 6.8 Hz, 3H, Me-1 Val), 0.98 (d, *J* = 6.8 Hz, 3H, Me-2 Val).
^{13}C NMR (101 MHz; D_2O): δ 174.9 (COγ Glu), 174.1 (CO Val), 173.49 (CO Gly), 173.40 (COα Glu), 59.6 (Cα Val), 53.8 (Cα Glu), 41.3 (Cα Gly), 31.1 (Cγ Glu), 29.9 (Cβ Val), 26.1 (Cβ Glu), 18.3 (Me-1 Val), 17.3 (Me-2 Val).
ESI-MS, negative mode: m/z 302.56 $[M - H]^-$; 324.55 $[M - 2H + Na]^-$

References

1. C. F. Morelli, C. Calvio, M. Biagiotti, and G. Speranza, *FEBS J.,* 2014, **281,** 232–245.
